# Beneficial effects of carvacrol loaded phytosomes on enhancing cryotolerance of Buffalo semen following cryopreservation

**DOI:** 10.1038/s41598-025-10030-0

**Published:** 2025-07-14

**Authors:** Wael A. Khalil, Salwa A. Elkhamy, Mohamed M Hegazy, Mahmoud A. E. Hassan, Ahmed Mowafy Tafish, Sameh A. Abdelnour, Mostafa A. El-Harairy

**Affiliations:** 1 Department of Animal Production, Faculty of Agriculture, Mansoura University, Mansoura, 35516 Egypt; 2 Animal Production Research Institute, Agriculture Research Centre, Ministry of Agriculture, Dokki, Giza 12619 Egypt; 3 Department of Pharmaceutical Technology, Faculty of Pharmacy, Horus University, New Damietta, 34518 Egypt; 4 Department of Animal Production, Faculty of Agriculture, Zagazig University, Zagazig, 44511 Egypt

**Keywords:** Buffalo semen, Carvacrol-loaded phytosomes, Mitochondrial function, Redox Status, Microbiota, Molecular docking, Developmental biology, Zoology

## Abstract

Sperm cryopreservation technology underpins genetic advancement in animal breeding and ongoing research aims to minimize cryoinjuries. This study aimed to develop carvacrol-loaded phytosomes (CLNPs) to enhance the physicochemical properties of carvacrol in aqueous cryopreservation extenders. Semen samples from five buffalo bulls were collected, extended and cryopreserved with varying CLNPs concentrations (0, 2.5, 5, 10, and 20 µg/mL). The freshly prepared CLNPs exhibited an average particle size of 286.7 ± 11.27 nm, a polydispersity index of 0.189 ± 0.05, and a zeta potential of − 11.4 ± 0.26 mV. Supplementing the freezing media with CLNPs significantly enhanced sperm progressive motility, viability, and plasma membrane integrity after both equilibration (5 °C for 4 h) and thawing. Furthermore, sperm kinematic parameters were significantly higher in all CLNPs-treated groups (*P* < 0.05). Compared to the CLNPs-free extender, CLNPs supplementation significantly reduced the percentage of dead sperm with intact acrosomes and increased the percentage of live sperm with intact acrosomes (*P* < 0.001). Post-thaw oxidative stress markers, including H_2_O_2_ and MDA, were significantly lower in all CLNPs groups (*P* < 0.001). Notably, the addition of 10 or 20 µg/mL of CLNPs increased TAC and significantly decreased nitric oxide levels compared to the control. Mitochondrial membrane potential and viable sperm counts were significantly higher in the CLNPs-treated groups (*P* < 0.001). CLNPs also significantly decreased the total bacterial count, spore-forming bacteria, and *coliform* bacteria in the post-thawed semen microbiota (*P* < 0.001). Higher CLNPs concentrations (10 or 20 µg/mL) appeared to provide superior protection, as evidenced by a greater proportion of sperm cells displaying normal nuclear, plasma membrane, mitochondrial, and acrosomal morphology. The pregnancy rate in the 20 µg/mL CLNPs group (86%, n = 43/50) was higher than in the control group (72%, n = 36/50). Molecular docking analysis revealed binding energies of − 6.22, − 4.93, − 4.44, and − 5.36 kcal/mol for Cox7c, Hsp70, PrxIII, and ATP1B1, respectively. This study introduces a novel nanotechnology-based approach using CLNPs to enhance buffalo semen cryopreservation, potentially significantly advancing assisted reproductive technologies in buffalo.

## Introduction

The water buffalo (*Bubalus bubalis*) is a crucial livestock species, significantly contributing to global milk and meat production and supporting agricultural sustainability^1^. Artificial insemination (AI), a widely adopted reproductive biotechnology, has revolutionized livestock breeding by enhancing productivity and accelerating genetic improvement^2^. Essential for the continuity, sustainability, and commercial viability of water buffalo farming in numerous countries^1,2^, AI relies heavily on semen cryopreservation. While semen cryopreservation techniques in buffaloes have advanced considerably in the past two decades and serve as a primary strategy for accelerating genetic progress in milk and meat production^3,4^, further optimization is necessary to mitigate the detrimental effects of the process^5,6^. Buffalo spermatozoa are particularly susceptible to cryopreservation-induced oxidative stress^5^, which can impair sperm motility, viability, membrane integrity, and DNA integrity^7^, ultimately compromising fertility^4,8^. Oxidative stress (OS) arises from an imbalance between reactive oxygen species (ROS) generation and antioxidant capacity during semen preservation and is a major contributor to the reduced quality of cryopreserved sperm. Supplementation of cryopreservation extenders with exogenous antioxidants^4^ may effectively counteract OS by interrupting the oxidative chain reaction.

Phytochemicals, particularly polyphenolic compounds, exhibit promising biological activities, including antioxidant and anti-apoptotic effects^9^. Carvacrol (CAR), a well-known phenolic compound abundant in aromatic plants of the *Origanum* and *Thymus* genera, has been extensively explored for its various properties^10^. However, CAR’s poor physicochemical properties, such as low bioavailability, stability, and high volatility, limit its potential pharmacological applications^11,12^. Rapidly advancing nanotechnology approaches offer site-specific delivery in a controlled manner at the nanoscale. This has generated significant interest due to their potential to enhance bioavailability, reduce side effects, and bypass first-pass metabolism. Various nanocarriers, including nanoliposomes^5^, nanoemulsions^13^, and nanoparticles^6,14^, have been applied to enhance semen preservation. While we previously explored the use of nanoliposomes in this context, phytosomes has emerged as promising alternatives.

Phytosomes are complexes formed by the interaction of phytoconstituents and phospholipids. Liposomes, synthetic vesicles mimicking natural membranes, are extensively studied and utilized nanocarriers with a well-established safety profile. Also known as herbosomes, phytosomes have garnered increasing interest in their potential to enhance the bioavailability of poorly soluble plant extracts and phytocompounds^15^. Compared to conventional herbal extracts, phytosomes demonstrate improved bioavailability, enhanced stability, and increased membrane permeability. As pharmaceutical formulations designed to encapsulate natural compounds or extracts, phytosomes function as effective herbal delivery systems^16^. These complexes, formed through hydrogen bonding interactions between biocompatible phospholipids and target active compounds^17,18^, leverage the essential role of phospholipids in maintaining the structural integrity of sperm cell membranes^19^.

Carvacrol (CAR) possesses diverse therapeutic potential, including antibacterial and antioxidant properties. Consequently, strategies to enhance its therapeutic application are urgently needed. Phytosome conjugation of carvacrol may yield novel delivery systems that overcome its low bioavailability, improve efficacy, and minimize adverse effects^12^. Notably, carvacrol in phytosome form exhibits superior benefits compared to liposomal formulations^20^. Phytosomes offer improved drug delivery compared to liposomes by chemically binding active compounds, enhancing stability and bioavailability^20^. Unlike liposomes, which are prone to leakage due to weak compound association, phytosomes utilize a phospholipid matrix, making them effective for poorly soluble molecules^18^. Fabrication methods include supercritical fluid and solvent-based techniques^21^. Phytosome-conjugated carvacrol has been utilized as a growth promoter in the broiler industry^22^ and demonstrates potential as a therapeutic strategy for wound healing^12^. While carvacrol has been investigated for its ability to alleviate OS, improve antioxidant status and cryopreserved sperm quality in human^23^, and goat semen preserved at 4 °C for 48 h^24^, the supplementation of CLNPs to buffalo semen cryopreservation media remains unexplored. This study aimed to evaluate the potential benefits of CLNPs on cryopreserved buffalo semen, including assessments of sperm quality, kinematics, redox homeostasis, microbial populations, apoptosis, acrosome integrity, sperm ultrastructure, in silico molecular docking and in vivo fertilization capacity.

## Material and methods

### Materials sources and ethical approval

Carvacrol was obtained from Sigma-Aldrich Co. (USA), and Phospholipon 90H (saturated soybean phosphatidylcholine) was generously supplied by Lipoid GmbH (Germany). Absolute ethanol was purchased from El-Nasr Pharmaceutical Chemicals Co. (Egypt). All experimental practices and protocols adhered to Directive 2010/63/EU of the European Parliament and Council of 22 September 2010 regarding the protection of animals used for scientific purposes. These practices were authorized by the Mansoura University Animal Care and Use Committee (Code number: MU-ACUC AGR.PhD.24.09.1) and conducted in accordance with the ARRIVE guidelines 2.0. Furthermore, the experimental protocols were approved and conducted following the guidelines and regulations of the Animal Care and Use Committee of Mansoura University.

### Preparation of carvacrol-loaded phytosomes

Carvacrol was formulated into phytosomes using the thin film hydration technique^12^. Briefly, equimolar amounts of carvacrol and Phospholipon 90H (1:1 molar ratio) were dissolved in 10 mL of absolute ethanol under magnetic stirring. This mixture was then refluxed in a 500 mL round-bottom flask under vacuum using a rotary evaporator (Wheaton, Chicago, Illinois, USA) at 45 °C and 110 rpm to form a thin film on the flask’s inner wall. Following complete ethanol evaporation, the resulting film was hydrated with 10 mL of distilled water to obtain the phytosomal suspension. This suspension was homogenized using a probe sonicator (SONICS Vibra-cell, Sonic & Materials, INC, USA) at 65% amplitude for 3 min in an ice bath. The freshly prepared phytosomal formulation was appropriately diluted and characterized for particle size (PS), polydispersity index (PDI), and zeta potential (ZP) using a Zetasizer (ZEN 3600, Malvern Instruments Limited Co., UK). Additionally, the morphology of the freshly prepared phytosomal formulation was examined using transmission electron microscopy (TEM; JOEL 2100, Japan).

### Animals and semen collection

Five mature, healthy water buffalo bulls (*B. bubalis*), and aged 4–6 years were included in this study at Mahat Moussa. These bulls were housed and fed according to standardized diets and management practices according to the International Livestock Management Training Center (ILMTC) in Kafr El-Sheikh Governorate, Egypt. Routinely used for semen collection at the center, these bulls belonged to the Egyptian buffalo breed. Semen samples were collected weekly over a 10-week period (total of 50 ejaculates) using an artificial vagina (pre-warmed to 42 °C) and immediately transported to the laboratory^25^. Two qualified investigators assessed each ejaculate for sperm motility using a phase contrast microscope (100X). Ejaculates meeting the standard minimum criteria for progressive motility and viability (> 75%), abnormality (< 15%), and sperm cell concentration (80 × 10^7^/mL)^5^ were accepted for experimental procedures. To minimize individual bull variability, ejaculates from all five bulls were pooled weekly on the day of collection, forming a single homogenized sample. Only five ejaculates were excluded from the entire study for not meeting quality criteria (e.g., motility, viability), leaving nine pooled samples for analysis. Subsequently, the accepted semen samples were pooled and prepared for the experiment.

### Extender preparation, experimental design and freezing and thawing

Following standard procedures at ILMTC and consistent with our previously published work^6,14^, the semen extender was prepared with minor adjustments to the formula described by Khalil et al.^26^. The Tris-citric-egg yolk extender consisted of citric acid (1.675 g), glycerol (6 mL), Tris (3.028 g), egg yolk (20 mL), fructose (1.25 g), penicillin (100 IU/mL), and streptomycin (100 µg/mL) in double distilled water, with the final volume adjusted to 100 mL. Before adding cryoprotectants, the extender’s osmolality was measured to be 280–300 mOsmol using a micro-osmometer (Berlin, Germany), and its pH was 6.8–6.9 using a pH meter. Prior to semen extension, the extender was gently mixed and warmed to 37 °C in a water bath.

The Tris-citric-egg yolk freezing extender served as the control group. Four treatment groups consisted of this base extender supplemented with 2.5 (CLNPs2.5), 5 (CLNPs5), 10 (CLNPs10), and 20 (CLNPs20) µg/mL of carvacrol-loaded phytosomes (CLNPs). Semen was extended at a 1:10 ratio (semen:extender) to achieve a final sperm concentration of 8 × 10⁷ sperm/mL in the diluted semen. Post-dilution, semen was gently agitated and equilibrated at 5 °C for 4 h. Subsequently, it was loaded into 0.5 mL straws (IVM Technologies, France), cooled over 4 cm of liquid nitrogen vapor for 10 min, and then plunged into liquid nitrogen for cryopreservation (4 weeks). Thawing was performed in a 37 °C water bath for 30 s. Semen samples were evaluated at three time points: after equilibration (5 °C, 4 h), post-thaw (37 °C, 30 s), and following incubation at 37 °C and 5% CO_2_ for 2 h.

### Evaluation of sperm quality

Sperm progressive motility was assessed at the previously mentioned time points using a phase-contrast microscope (Leica DM 500, Switzerland) equipped with a 37 °C hot stage. The detailed methodology for motility assessment can be found in our earlier work^6^. Sperm viability was determined using the one-step eosin-nigrosin staining technique^27^. For viability and morphology assessment, 10 µL aliquots of each sample were placed on slides, stained with eosin-nigrosin, and examined under a light microscope (400 × magnification). At least 200 spermatozoa were evaluated per slide, with live spermatozoa appearing unstained (white) and dead spermatozoa exhibiting red coloration in the head. The incidence of tail and head abnormalities was also recorded.

Sperm plasma membrane integrity was evaluated using the hypo-osmotic swelling test (HOST) at 75 mOsmol/L^14^. Briefly, 50 µL of diluted semen was mixed with 500 µL of HOST solution and incubated at 37 °C for 30 min. A 10 µL aliquot of this mixture was then examined under a phase contrast microscope, with at least 200 spermatozoa evaluated. Spermatozoa displaying swollen or coiled tails were considered to have intact plasma membranes, as previously described.

### Sperm kinematics using CASA

The sperm kinematic parameters were assessed using a CASA system (Sperm Vision, Ref: 12,520/5000; Minitube Hauptstrae 41. 84,184 Tiefenbach, Germany), according to the methodology outlined in^25^. CASA was employed to analyze approximately 1500 spermatozoa per treatment at 37 °C. Sperm motility was visualized using an Olympus BX microscope (Hamburg, Germany) under × 4 dark-field illumination, with images captured at 60 Hz (60 frames per second) by a rapid scan digital camera connected to the microscope. This system employs a computer-aided microscope (Olympus) to capture real-time sperm images and analyze their motility characteristics. The following sperm kinematic parameters were evaluated: distance straight line (DSL, µm), distance average path (DAP, µm), distance curved line (DCL, µm), velocity average path (VAP, µm/sec), velocity curved line (VCL, µm/sec), straightness (STR = VSL/VAP), velocity straight line (VSL, µm/sec), linearity (LIN = VSL/VCL), wobble (WOB = VAP/VCL), beat cross frequency (BCF, Hz), and amplitude of lateral head displacement (ALH, µm).

### Estimation of acrosome integrity

Acrosome integrity was assessed as detailed in our previous publications^6^. Briefly, 200 µL of post-thawed semen was incubated with 0.2% trypan blue at 37 °C for 10 min. Spermatozoa were then diluted with albumin-free modified Brackett and Oliphant (mBO) medium and centrifuged at 700 g for 6 min, following established procedures^28^.

Sperm pellets were washed by repeated centrifugation and supernatant removal until clear or pale blue, with resuspension in the original volume after each wash. A 10–20 µL aliquot of the washed sperm suspension was smeared onto a glass slide, air-dried at 40 °C, and stained with a freshly prepared 10% Giemsa solution for 45 min. Slides were rinsed with distilled water and air-dried. Acrosome integrity was evaluated by bright-field microscopy at 1000X magnification, examining approximately 100 randomly selected spermatozoa per slide, based on the following criteria;

- Viable, Intact Acrosome: Post-acrosome white, acrosome bright pink/purple.
- Dead, Intact Acrosome: Post-acrosome blue/dark blue, acrosome dark pink/purple.
- Live, Detached Acrosome (True Acrosome Reaction): Post-acrosome white, acrosome white.
- Dead, Detached Acrosome (False Acrosome Reaction): Post-acrosome blue, acrosome white/gray.

### Apoptosis like changes using flow cytometry

Apoptosis in three semen samples per treatment group was evaluated using Annexin V/PI staining, following a previously established protocol^29^. Briefly, 1 mL of semen was resuspended in 2 mL of binding buffer and incubated in the dark at room temperature for 15 min with 5 μL of Annexin V-FITC and 5 μL of propidium iodide (PI). After incubation, samples were diluted with 200 μL of binding buffer and analyzed using an Accuri C6 flow cytometer (BD Biosciences) and its software, as detailed elsewhere^30^. The percentages of positive or negative Annexin V (A − /A +), PI (PI − /PI +), and the double-positive cells were assessed. According to Peña et al.^31^, sperm were categorized into four classes:

- Viable cells: without fluorescence signal and membrane dysfunction (A − /PI −).
- Early apoptotic sperm cells: viable cells labeled with Annexin V but without PI (A + /PI −).
- Necrotic sperm cells: non-viable cells labeled with PI without Annexin V and with complete membrane loss (A − /PI +).
- Apoptotic sperm cells: Non-viable cells labeled with Annexin V and PI and with damaged permeable membranes (A + /PI +). Lastly, the number of spermatozoa in each previous category was recorded in each group. Cell populations were identified based on their forward and side scattered properties^31^.

### Mitochondrial membrane potential

Sperm mitochondrial activity was assessed using the lipophilic cation JC-10 (JC-10 Mitochondrial Membrane Potential Assay Kit (Flow Cytometry) ab112133), following the methodology described in previous study^32^. The resulting pellet was resuspended in 500 μL of JC-10 and incubated for 1 h at 37 °C. After centrifugation, cells were resuspended in PBS and analyzed by flow cytometry using a 595 nm emission filter. The percentage of spermatozoa exhibiting orange fluorescence (JC-10 aggregates), indicative of high mitochondrial membrane potential (HMMP), was quantified.

### Assessment of oxidative stress of post-thawed buffalo bull semen

Following thawing, semen samples (n = 5 per treatment group) were centrifuged at 1600*g* for 10 min. The supernatant extender was then carefully separated and stored at − 20 °C for subsequent analysis. The concentrations of total antioxidant capacity (TAC), malondialdehyde (MDA), hydrogen peroxide (H_2_O_2_), and nitric oxide (NO) were determined in these post-thaw media. The assays for TAC, MDA, H_2_O_2_, and NO were performed using commercially available colorimetric kits provided by Biodiagnostic Company (Giza, Egypt), strictly following the protocols outlined in^33–36^, respectively, and in accordance with the manufacturer’s instructions.

### Microbiological analysis of semen samples

Total bacterial counts in the examined post-thawed semen samples were performed following the method described in^37^, utilizing nutrient agar medium^38,39^. Plates were incubated at 37 °C for 24 h, after which colony-forming units (CFU) were enumerated. Aerobic spore-forming bacteria were quantified by initially subjecting the semen samples to a heat treatment of 80 °C for 10 min, followed by cooling to 30–32 °C. These treated samples were then serially diluted, plated onto nutrient agar medium, and incubated at 30 °C for 48 h before counting CFU. The detection and enumeration of *coliform* bacteria in the examined semen samples were conducted according to the methodology outlined in^40^. Plates were incubated at 37 °C for 16–18h prior to counting. All microbiological analyses were performed on six independent semen samples per treatment group.

### Spermatozoa ultrastructure using TEM

For ultrastructural analysis, three post-thawed semen samples per group were processed for TEM. The preparation method was a slight modification of that described in^26^. Briefly, semen samples were fixed in 2% glutaraldehyde in PBS for 2–3 h, washed three times with PBS by centrifugation at 4 °C, and then subjected to post-fixation in 1% osmium tetroxide for approximately 2 h at 4 °C. Following fixation, the sperm were dehydrated using an acetone gradient, embedded in Epon resin, and sectioned into ultrathin slices using an RMC ultramicrotome. The resulting sections were stained with uranyl acetate and lead citrate. TEM observation of random fields was performed using a microscope equipped with an AMT Optronics CCD camera.

### Exo-vivo experiment

A total of 100 multiparous buffalo cows, synchronized for spontaneous estrus, were randomly allocated to two groups (n = 50 per group). The control group (Group 1) was artificially inseminated with frozen-thawed control semen. The treatment group received artificial insemination with frozen-thawed semen treated with 20 µg/mL CLNPs, this concentration having demonstrated the most favorable outcome in the in vitro analyses. All cows received a single artificial insemination. Pregnancy success was evaluated using the 56-day non-return rate^6^.

### In silico analyses

Carvacrol was subjected to active site molecular docking simulation with four different targeted proteins. Carvacrol’s structure was acquired from the PubChem database (pubchem.ncbi.nlm.nih.gov). Open Babel software (version 2.3.2) was used to convert the SDF file format of carvacrol into PDBQT file format. Protein molecular targets heat shock protein 70 (Hsp70, ID: 7O6R), Mitochondrial 2-cys peroxiredoxin III, (PrxIII, ID: 4MH3), cytochrome c oxidase subunit 7C (COX7C, ID: 6FF5), and ATPase Subunit Beta-1 (ATP1B1, ID: 4XE5) were downloaded from the Protein Data Bank (PDB: www.rcsb.org.) Following protein pretreatments, including eliminating water molecules and hetatoms, were done using Biovia Discovery Studio software. The cofactors of the selected proteins remained to uphold their function. The SWISS-PDBVIEWER (SPDBV) version 4.1.0 was employed to both minimize energy and verify the presence of any absent components in the selected receptors. Autodock 4.2 software fulfilled the molecular docking analysis. The procedure starts by integrating polar hydrogens into the protein, then progresses to applying Kollman charges as well as Gasteiger charges. The grid box was designed to encompass the active site region of each protein, where for 7O6R it was located at (X = − 5.442, Y = − 14.810, Z = − 16.365), for 4MH3 it was (X = 64.287, Y = − 23.949, Z = 47.584), for 6FF5 it was (X = 2.848, Y = − 12.610, Z = − 16.072), and (X = − 5.089, Y = − 39.867, Z = − 34.595) for 4XE5. The docking features were adapted to genetic algorithms, resulting in the detection of 50 conformations/poses. Subsequently, the best conformer was selected based on the least inhibition constant Ki as well as the least free energy of binding. The LigPlus (LigPlot + v.2.2.8) software was employed to identify the hydrophobic interactions between carvacrol and the targeted protein. Additionally, Biovia Discovery Studio Visulaizer version 4.5 (https://discover.3ds.com/discovery-studio-visualizer-download) was employed for the visualization of the best-posed conformers.

### Statistical analysis

Data are presented as mean ± standard error (SE). Prior to statistical analysis, the normality of data distribution was assessed using the Shapiro–Wilk test, and the homogeneity of variances was evaluated using Levene’s test. The effect of treatment was analyzed using one-way analysis of variance (ANOVA) in IBM SPSS Statistics version 25. The following mathematical model was employed to explore all measurements, Yij = μ + TRTi + eij, where Yij = observations, μ = overall mean, TRT = effect of the CLNPs (i, 1–5), and eij = random error. The pregnancy rate was statistically analyzed using the chi-square test (χ^2^). The significant differences were accomplished by Tukey’s test at *P* < 0.05.

## Results

### Characterization of carvacrol-loaded phytosomes

**Fig. 1:**
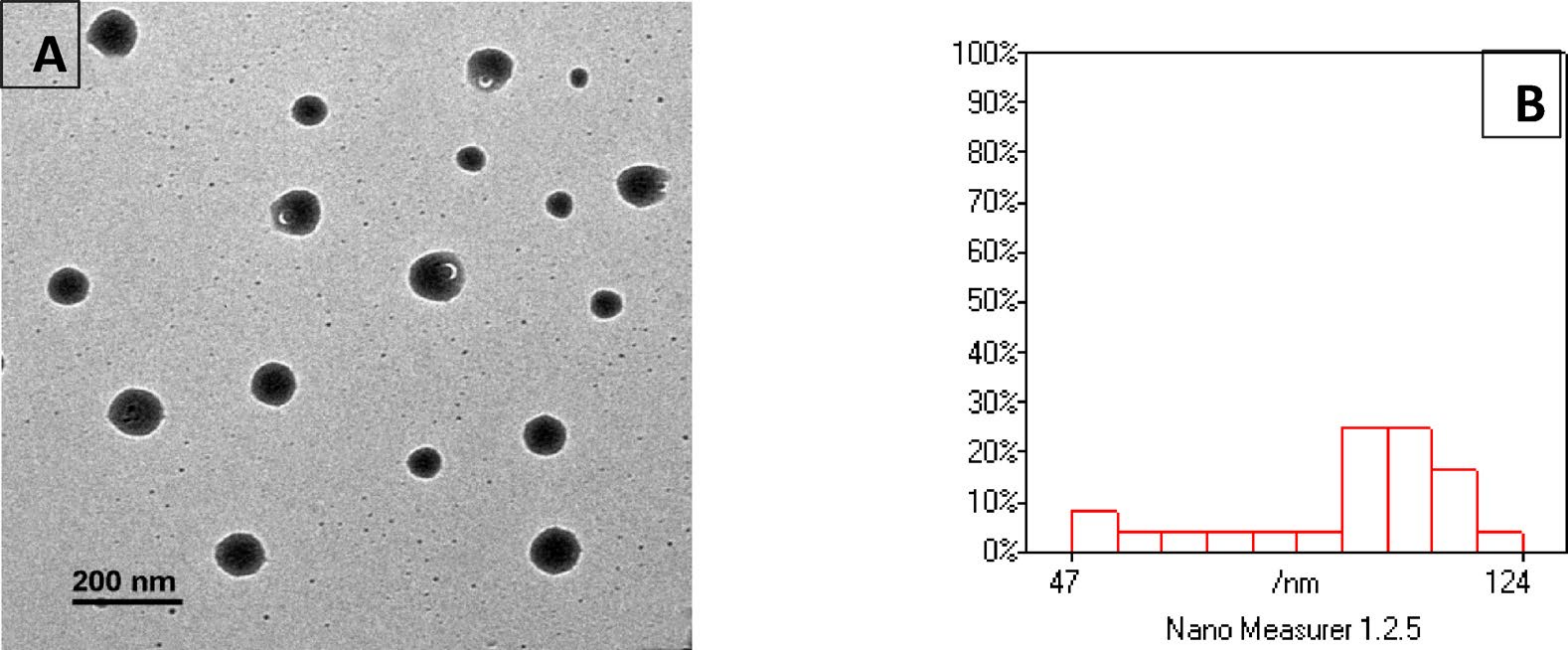
(**A**) The morphology of carvacrol-loaded phytosomes nanoparticles by TEM showing vesicle-like entities with spherical shapes could be proved. Additionally, a uniform size distribution without agglomeration was noticed, (**B**) histogram shows that most particles cluster around 47–124 nm, with a good distribution.

The freshly prepared carvacrol-loaded phytosomal formulation exhibited an average particle size (PS) of 286.7 ± 11.27 nm, a polydispersity index (PDI) of 0.189 ± 0.05, and a zeta potential (ZP) of − 11.4 ± 0.26 mV. Morphological analysis of the phytosomal suspension via TEM revealed spherical nanoparticles with no evidence of aggregation (Fig. 1).

### Impacts of CLNPs on buffalo bull semen after equilibration (5 °C for 4 h)

Following 4 h of equilibration at 5 °C, sperm abnormality remained unchanged (*P* = 0.85). The additive of CLNPs to the extender significantly enhanced membrane integrity (*P* = 0.01). Moreover, supplementing the extender with 20 µg/mL CLNPs significantly improved (*P* < 0.05) sperm viability and progressive motility compared to the CLNPs-free control (CLNPs0), with no significant differences observed among other CLNPs concentrations (*P* > 0.05). CLNPs concentrations up to 10 µg/mL did not significantly affect sperm viability (*P* > 0.05) or progressive motility (*P* > 0.05) compared to the CLNPs-free extender (Table 1).

**Table 1:** Impact of carvacrol-loaded phytosome-fortified freezing extender on buffalo sperm characteristics following equilibration at 5 °C for 4 h (Mean ± SE, n = 9).

Extender	Sperm characteristics (%)			
	Progressive motility	Viability	Membrane integrity	Abnormality
Control	71.7 ± 1.67^b^	73.0 ± 1.53^b^	71.3 ± 1.20^b^	7.0 ± 0.58
CLNPs (2.5 µg/mL)	80.0 ± 2.89^ab^	81.3 ± 3.28^ab^	82.7 ± 2.73^a^	8.0 ± 1.53
CLNPs (5.0 µg/mL)	80.0 ± 2.89^ab^	84.3 ± 3.28^ab^	83.3 ± 3.67^a^	6.7 ± 1.76
CLNPs (10 µg/mL)	81.7 ± 1.67^ab^	84.7 ± 2.19^ab^	84.7 ± 1.20^a^	6.0 ± 1.00
CLNPs (20 µg/mL)	83.3 ± 1.67^a^	85.0 ± 2.08^a^	85.7 ± 1.45^a^	7.0 ± 1.00
*P* value	0.03	0.04	0.01	0.850

^a-b^Means denoted with different superscripts in each column are significantly different at *P* < 0.05.

### Impacts of CLNPs on post-thawed buffalo bull semen

After cryopreservation, the results indicate that the addition of CLNPs to the buffalo semen freezing media led to significantly greater progressive motility, viability, and membrane integrity compared to the CLNPs-free extender (*P* < 0.001). In contrast, the presence of CLNPs in the freezing media did not significantly alter buffalo sperm abnormalities (*P* = 0.73). The freezing extender supplemented with 20 µg/mL CLNPs yielded the most favorable results for sperm viability and progressive motility. Furthermore, a gradual improvement in sperm progressive motility was noted with increasing concentrations of CLNPs in the buffalo semen freezing diluent (Table 2).

**Table 2:** Impact of carvacrol-loaded phytosome-fortified freezing extender on sperm characteristics of post-thawed buffalo bull semen at 37 °C for 30 s (Mean ± SE, n = 9).

Extender	Sperm characteristics (%)			
	Progressive motility	Viability	Membrane integrity	Abnormality
Control	36.1 ± 0.73^d^	38.8 ± 1.12^c^	37.9 ± 1.14^b^	10.6 ± 0.65
CLNPs (2.5 µg/mL)	43.3 ± 1.18^c^	45.0 ± 1.18^b^	45.4 ± 1.26^a^	9.6 ± 0.73
CLNPs (5.0 µg/mL)	43.9 ± 1.11^bc^	45.1 ± 1.54^b^	46.0 ± 1.67^a^	9.2 ± 0.92
CLNPs (10 µg/mL)	47.8 ± 0.88^ab^	49.7 ± 1.13^ab^	48.7 ± 1.34^a^	9.9 ± 0.87
CLNPs (20 µg/mL)	48.9 ± 1.11^a^	51.1 ± 0.99^a^	50.0 ± 1.00^a^	10.4 ± 0.78
*P* value	< 0.0001	< 0.0001	< 0.0001	0.730

^a-d^Means denoted with different superscripts in each column are significantly different at *P* < 0.05.

### Impacts of CLNPs on kinematic parameters of post-thawed buffalo bull sperm

CASA is a widely applied technique for evaluating sperm quality in diverse animal species and humans, enabling the assessment of various sperm motion parameters. The results demonstrated that the addition of CLNPs to the freezing extender significantly improved most buffalo sperm kinematic parameters (except for WOB and ALH) (*P* < 0.05). Notably, the most pronounced effect on sperm motion parameters was observed with the addition of 10 or 20 µg/mL of CLNPs compared to other treatment groups (Table 3).

**Table 3:** Impact of carvacrol-loaded phytosome-fortified freezing extender on kinematic parameters of post-thawed buffalo bull sperm (Mean ± SE, n = 7).

Item	Control	CLNPs (2.5 µg/mL)	CLNPs (5.0 µg/mL)	CLNPs (10 µg/mL)	CLNPs (20 µg/mL)	*P* value
PM (%)	37.6 ± 1.36^c^	42.9 ± 0.90^bc^	47.0 ± 1.28^b^	52.7 ± 0.90^a^	54.0 ± 2.02^a^	< 0.0001
DAP (µm)	15.3 ± 0.37^b^	18.0 ± 0.67^a^	20.4 ± 0.51^a^	20.1 ± 1.08^a^	20.6 ± 0.43^a^	< 0.0001
DCL (µm)	22.1 ± 0.78^b^	27.1 ± 1.16^a^	30.0 ± 0.80^a^	29.2 ± 1.44^a^	29.8 ± 0.79^a^	< 0.0001
DSL (µm)	10.3 ± 0.25^c^	13.5 ± 0.49^b^	15.2 ± 0.40^ab^	16.1 ± 0.85^a^	16.3 ± 0.34^a^	< 0.0001
VAP (µm/sec)	35.9 ± 0.85^b^	40.4 ± 1.59^ab^	45.4 ± 1.26^a^	44.6 ± 2.19^a^	45.1 ± 0.92^a^	0.0002
VCL (µm/sec)	51.9 ± 1.43^b^	60.6 ± 2.75^ab^	66.5 ± 1.90^a^	67.2 ± 4.04^a^	65.1 ± 1.85^a^	0.001
VSL (µm/sec)	24.6 ± 0.77^c^	30.3 ± 1.11^b^	33.8 ± 0.91^ab^	35.8 ± 1.74^a^	35.7 ± 0.56^a^	< 0.0001
STR (%)	67.7 ± 1.69^c^	74.6 ± 1.09^b^	74.0 ± 1.18^b^	80.0 ± 1.35^a^	78.9 ± 0.91^ab^	< 0.0001
LIN (%)	47.0 ± 2.02^b^	49.7 ± 0.81^ab^	50.6 ± 1.00^ab^	53.3 ± 1.80^a^	54.6 ± 1.21^a^	0.01
WOB (%)	69.0 ± 1.53	66.3 ± 0.64	67.7 ± 0.78	66.3 ± 1.39	68.9 ± 0.77	0.23
ALH (µm)	2.4 ± 0.11	2.4 ± 0.05	2.5 ± 0.07	2.4 ± 0.18	2.6 ± 0.11	0.62
BCF (Hz)	19.0 ± 0.49^c^	24.8 ± 0.37^ab^	26.6 ± 0.71^ab^	28.7 ± 1.61^a^	28.3 ± 0.96^ab^	< 0.0001

PM, progressive motility; DCL, distance curved line (µm); DAP, distance average path (µm); DSL, distance straight line (µm); VCL, velocity curved line (µm/sec); VAP, velocity average path (µm/sec); VSL, velocity straight line (µm/sec); LIN, linearity (VSL/VCL); STR, straightness (VSL/VAP); WOB, wobble (VAP/VCL); BCF, beat cross frequency (Hz) and ALH, amplitude of lateral head displacement (µm).

^a-c^Means denoted with different superscripts in each column are significantly different at *P* < 0.05.

### Impacts of CLNPs on acrosome status

The addition of CLNPs to buffalo semen extender did not significantly affect the proportions of live sperm with detached acrosomes (*P* = 0.51) or dead sperm with detached acrosomes (*P* = 0.09) compared to the control group (Table 4). However, all CLNPs-supplemented extenders significantly reduced the proportion of dead sperm with intact acrosomes and significantly increased the proportion of live sperm with intact acrosomes compared to the CLNPs-free extender (*P* < 0.001). The 20 µg/mL CLNPs concentration resulted in the highest proportion of live sperm with intact acrosomes and the lowest proportion of dead sperm with intact acrosomes compared to other groups (*P* < 0.05).

**Table 4:** Impact of carvacrol-loaded phytosome-fortified freezing extender on acrosome status of post-thawed buffalo bull semen (Mean ± SE, n = 5).

Treatment	Acrosome Status (%)			
	Live sperm with intact acrosome	Live sperm with detached acrosome	Dead sperm with intact acrosome	Dead sperm with detached acrosome
Control	34.0 ± 1.00^c^	13.6 ± 0.93	48.8 ± 0.73^a^	3.6 ± 0.51
CLNPs (2.5 µg/mL)	43.0 ± 0.71^b^	12.4 ± 0.51	41.8 ± 0.58^b^	2.8 ± 0.37
CLNPs (5.0 µg/mL)	43.8 ± 0.86^b^	11.8 ± 0.86	40.8 ± 0.66^b^	3.6 ± 0.51
CLNPs (10 µg/mL)	44.8 ± 1.39^b^	11.8 ± 1.02	41.0 ± 1.10^b^	2.4 ± 0.24
CLNPs (20 µg/mL)	51.0 ± 1.30^a^	12.4 ± 0.51	34.2 ± 1.24^c^	2.4 ± 0.24
*P* value	< 0.0001	0.51	< 0.0001	0.09

^a-c^Means denoted within the same column with different superscripts are significantly different at *P* < 0.05.

### Impacts of CLNPs on semen incubated at 37 °C and 5% CO_2_ for 2 h

Cryopreserving buffalo semen with varying CLNPs concentrations significantly enhanced post-thaw sperm quality. Following a 2-h incubation at 37 °C and 5% CO_2_, CLNP-treated sperm exhibited significantly higher viability, progressive motility, and membrane integrity compared to control (*P* < 0.001, Table 5). Notably, the highest membrane integrity was achieved with 10 or 20 µg/mL CLNPs (*P* < 0.05). Furthermore, 20 µg/mL CLNPs resulted in superior progressive motility and viability compared to all other groups except the 10 µg/mL group (*P* < 0.05). No significant effects of CLNPs concentrations on sperm abnormalities were observed (*P* = 0.47).

**Table 5:** Impact of carvacrol-loaded phytosome-fortified freezing extender on post-thawed buffalo sperm characteristics following incubation at 37 °C and 5% CO_2_ for 2 h (Mean ± SE, n = 9).

Treatment	Sperm characteristics (%)			
	Progressive motility	Viability	Membrane integrity	Abnormality
Control	31.1 ± 0.73^d^	34.2 ± 1.15^c^	31.7 ± 0.78^c^	11.6 ± 0.60
CLNPs (2.5 µg/mL)	38.3 ± 1.18^c^	40.1 ± 1.24^b^	39.9 ± 1.09^b^	11.2 ± 0.52
CLNPs (5.0 µg/mL)	38.9 ± 1.39^bc^	41.3 ± 1.40^b^	40.2 ± 1.36^b^	10.1 ± 0.93
CLNPs (10 µg/mL)	42.8 ± 0.88^ab^	44.1 ± 0.75^ab^	44.3 ± 0.55^a^	11.1 ± 0.68
CLNPs (20 µg/mL)	43.3 ± 0.83^a^	45.8 ± 0.72^a^	45.0 ± 0.96^a^	11.7 ± 0.33
*P* value	< 0.0001	< 0.0001	< 0.0001	0.47

^a–d^Means denoted with different superscripts in each column are significantly different at *P* < 0.05.

### Impacts of CLNPs on redox status

MDA levels significantly decreased in buffalo semen extended with varying CLNPs concentrations (*P* < 0.001, Table 6). Supplementing the freezing medium with 10 or 20 µg/mL CLNPs significantly increased TAC levels and decreased NO levels compared to the free-CLNPs extender (*P* < 0.01). TAC and NO levels in the control group did not significantly differ from those in the 2.5 or 5.0 µg/mL CLNPs groups (*P* > 0.05). All CLNPs concentrations significantly reduced H_2_O_2_ levels compared to the control, with the lowest levels observed at 20 µg/mL CLNPs. Lower CLNPs concentrations (2.5, 5, and 10 µg/mL) exhibited similar effects on H_2_O_2_ levels (*P* > 0.05).

**Table 6:** Impact of carvacrol-loaded phytosome-fortified freezing extender on redox status of post-thawed buffalo bull semen (Mean ± SE, n = 5).

Treatment	TAC (mM/L)	MDA (nmol/mL)	H_2_O_2_ (mM/L)	NO (µmol / L)
Control	0.51 ± 0.05^b^	7.4 ± 0.34^a^	0.176 ± 0.005^a^	8.2 ± 0.35^a^
CLNPs (2.5 µg/mL)	0.56 ± 0.06^ab^	6.1 ± 0.20^b^	0.158 ± 0.004^b^	7.8 ± 0.40^a^
CLNPs (5.0 µg/mL)	0.63 ± 0.03^ab^	5.8 ± 0.21^b^	0.150 ± 0.005^bc^	7.0 ± 0.36^ab^
CLNPs (10 µg/mL)	0.72 ± 0.05^a^	5.7 ± 0.16^b^	0.146 ± 0.002^bc^	6.3 ± 0.29^b^
CLNPs (20 µg/mL)	0.74 ± 0.03^a^	5.3 ± 0.19^b^	0.138 ± 0.004^c^	6.0 ± 0.22^b^
*P* value	0.007	0.001	< 0.0001	< 0.0001

^a–c^Means denoted within the same column with different superscripts are significantly different at *P* < 0.05. Total Antioxidant concentration (TAC), malondialdehyde (MDA), hydrogen peroxide (H_2_O_2_) and nitric oxide (NO).

### Impacts CLNPs on apoptosis-like changes and mitochondrial membrane potential

The addition of CLNPs to the freezing medium significantly increased the percentage of viable sperm (*P* < 0.001), with the exception of the 2.5 µg/mL concentration (Table 7). The 20 µg/mL CLNP concentration resulted in the lowest percentage of apoptotic sperm (*P* < 0.001). The highest percentage of necrotic sperm was observed in the 5 and 10 µg/mL CLNP groups, while other groups showed comparable results (*P* > 0.05). Sperm mitochondrial membrane potential (MMP) was significantly enhanced by CLNPs supplementation, with the 20 µg/mL concentration showing the greatest improvement (*P* < 0.01). The 10 µg/mL CLNPs group exhibited intermediate MMP values compared to the 5 and 20 µg/mL groups (*P* > 0.05).

**Table 7:** Impact of carvacrol-loaded phytosome-fortified freezing extender on apoptosis-like changes and mitochondrial membrane potential (MMP) of post-thawed buffalo bull sperm (Mean ± SE, n = 3).

Treatment	Sperm characteristics (%)				
	Viable (A − /PI −)	Early apoptotic (A + /PI −)	Apoptotic (A + /PI +)	Necrotic (A − /PI +)	MMP
Control	21.4 ± 0.13^d^	0.30 ± 0.06^b^	74.8 ± 0.10^a^	3.5 ± 0.09^b^	11.8 ± 0.32^d^
CLNPs (2.5 µg/mL)	20.3 ± 0.59^d^	0.30 ± 0.06^b^	76.0 ± 0.64^a^	3.4 ± 0.06^b^	21.8 ± 1.91^c^
CLNPs (5.0 µg/mL)	45.3 ± 1.22^c^	0.10 ± 0.00^b^	42.1 ± 1.16^b^	12.5 ± 0.22^a^	38.0 ± 1.36^b^
CLNPs (10 µg/mL)	50.2 ± 1.54^b^	0.17 ± 0.17^b^	40.0 ± 2.11^b^	9.7 ± 2.85^a^	40.3 ± 0.64^ab^
CLNPs (20 µg/mL)	78.7 ± 0.77^a^	5.83 ± 2.32^a^	12.3 ± 1.55^c^	3.1 ± 0.40^b^	45.7 ± 1.79^a^
*P* value	< 0.0001	0.01	< 0.0001	0.001	< 0.0001

^a–d^means denoted within the same column with different superscripts are significantly different at *P* < 0.05.

### Impacts of CLNPs on semen microbiota

All CLNP-supplemented groups exhibited a significant reduction in total bacterial count, spore-forming bacteria, and *coliform* bacteria count (*P* < 0.001, Table 8). Spore-forming bacteria were significantly lower in all CLNPs groups compared to the CLNPs-free extender (*P* < 0.001). The lowest bacterial count post-cryopreservation was observed in the 20 µg/mL CLNPs group. The 5 µg/mL CLNPs group showed intermediate bacterial counts compared to the 2.5 and 10 µg/mL groups (*P* > 0.05).

**Table 8:** Impact of carvacrol-loaded phytosome-fortified freezing extender on semen microbiota load of post-thawed buffalo bull sperm (Mean ± SE, n = 6).

Treatment	Microbiota in Semen (CFUx10^3^ /mL)		
	Total bacterial count	Spore-forming bacteria	*Coliform* bacteria count
Control	398.3 ± 7.26^a^	3.3 ± 0.21^a^	336.7 ± 9.72^a^
CLNPs (2.5 µg/mL)	274.2 ± 9.08^b^	2.0 ± 0.18^b^	229.2 ± 8.60^b^
CLNPs (5.0 µg/mL)	248.3 ± 8.53^bc^	1.7 ± 0.25^b^	220.0 ± 10.25^bc^
CLNPs (10 µg/mL)	229.2 ± 10.99^c^	1.6 ± 0.24^b^	216.7 ± 7.92^bc^
CLNPs (20 µg/mL)	217.5 ± 8.64^c^	1.4 ± 0.20^b^	190.0 ± 8.16^c^
*P* value	< 0.0001	< 0.0001	< 0.0001

^a–c^means denoted within the same column with different superscripts are significantly different at *P* < 0.05.

#### Impacts of CLNPs on sperm ultrastructure

**Fig. 2:**
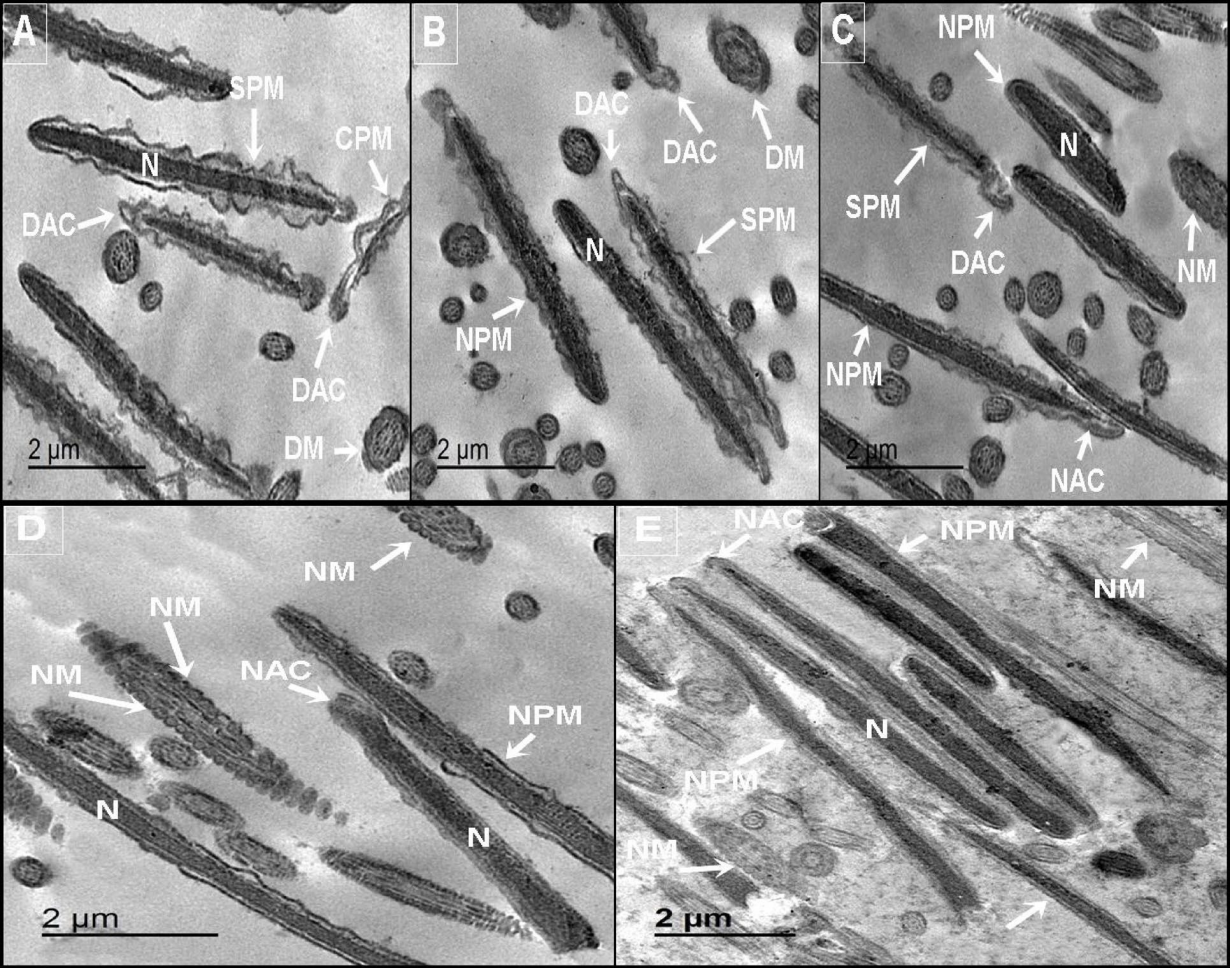
Impact of carvacrol-loaded phytosome on post-thawed buffalo sperm ultrastructural with various concentrations (**A**) 0, (**B**) 2.5 µg/mL, (**C**) 5.0 µg/mL, (**D**) 10 µg/mL and (**E**) 20 µg/mL. N (nucleus), CPM (complete damaged plasma membrane), SPM (slightly dilated plasma membrane), NPM (normal plasma membrane), NM (normal mitochondria), DM (damaged mitochondria), NAC (normal acrosomal), DAC (damaged acrosomal).

Figure 2A–E illustrates the ultrastructural effects of CLNPs at various concentrations on post-thawed buffalo sperm. The control group (Fig. 2A) exhibited several subcellular changes, including a completely damaged plasma membrane (CPM), slightly dilated plasma membrane (SPM), damaged acrosome (DAC), and damaged mitochondria (DM). Treatment with 2.5 µg/mL (Fig. 2B) or 5 µg/mL (Fig. 2C) CLNPs resulted in sperm displaying damaged mitochondria and acrosomes (DAC), a stable nucleus, and a slightly dilated plasma membrane, although some sperm retained a normal plasma membrane. In contrast, higher CLNPs concentrations of 10 µg/mL (Fig. 2D) and 20 µg/mL (Fig. 2E) appeared to provide a protective effect, with sperm cells showing a normal nucleus, normal plasma membrane, normal mitochondria (NM), and normal acrosome (NAC).

### Impacts of CLNPs on pregnancy rate

**Fig. 3:**
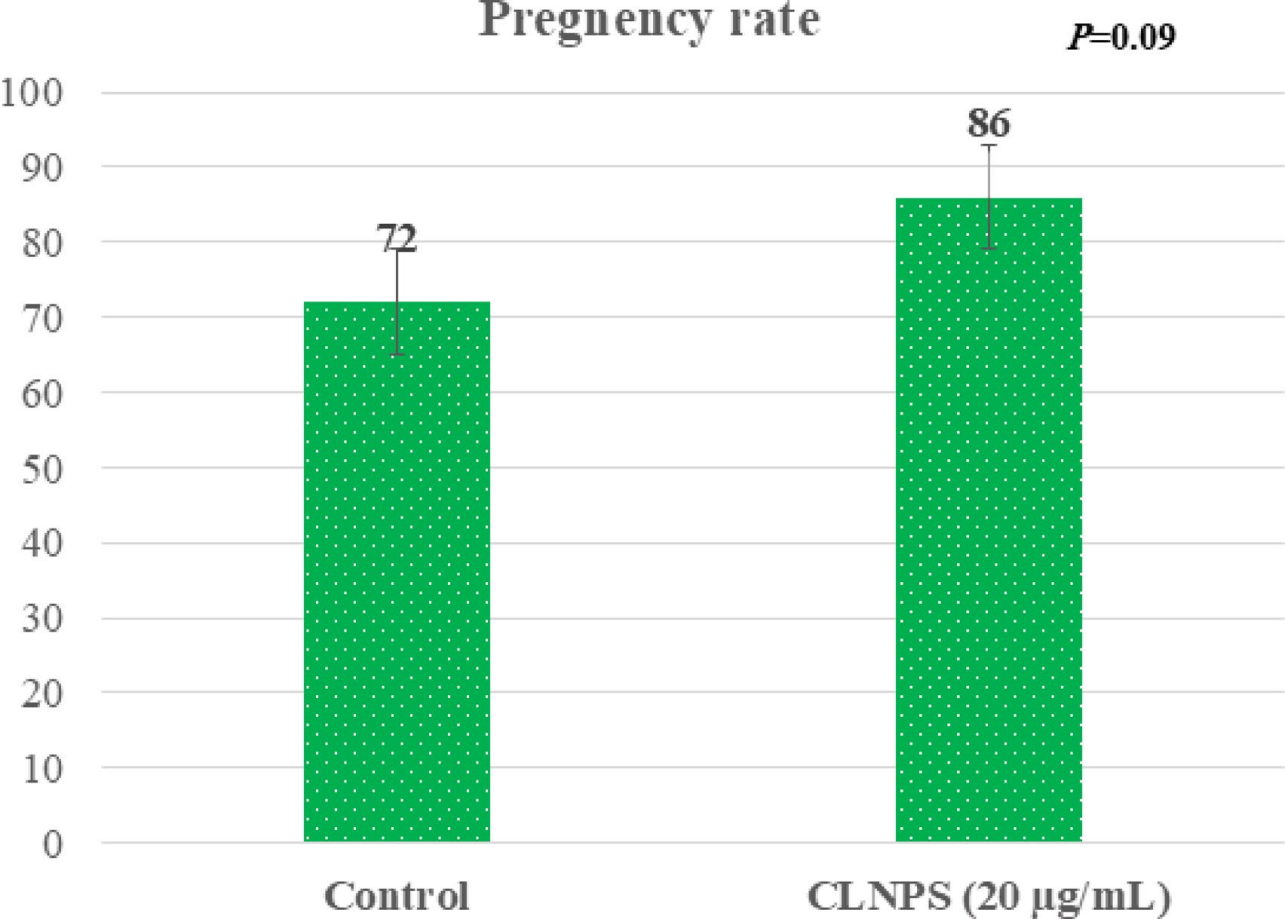
Effect of supplementing Tris-extender with carvacrol-loaded phytosomes (CLNPs, 20 µg/mL) on non-return rate (56 days) in buffalo cows.

Buffalo cows inseminated with the optimal CLNPs dose of 20 µg/mL showed a numerically higher pregnancy rate (86%, n = 43/50) compared to the control group (72%, n = 36/50) (Fig. 3).

### Molecular docking simulation

**Fig. 4:**
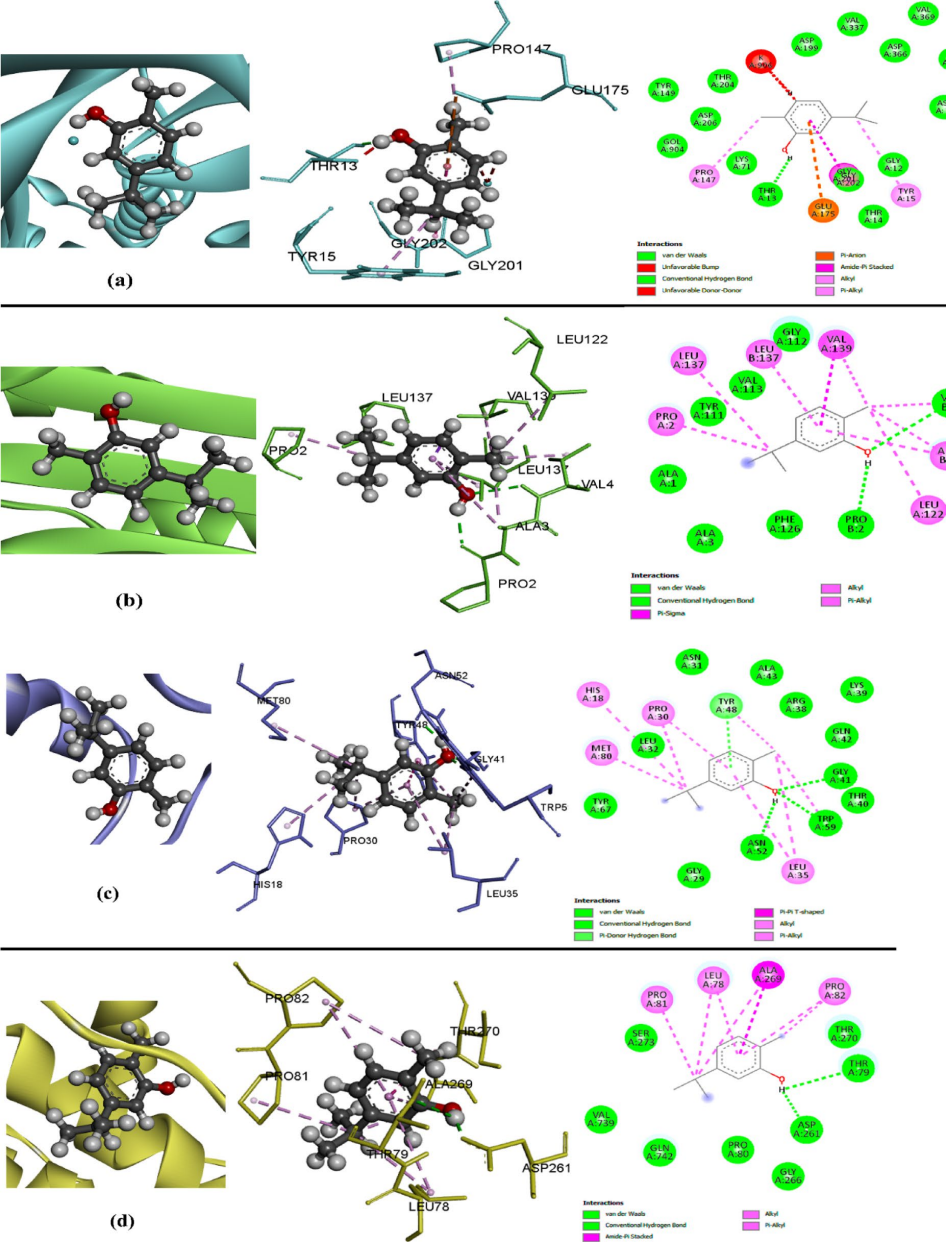
Images 2D and 3D of molecular docking binding between carvacrol and protein receptors Hsp70 (**a**), PrxIII (**b**), Cox7c (**c**) and ATP1B1 (**d**) in buffalo sperm.

Carvacrol, a potent phenolic monoterpenoid, exhibits a wide spectrum of bioactivities, including antimicrobial, antioxidant, and anticancer effects, making it a promising candidate for various clinical applications. This in silico study explores the association between carvacrol and protein receptors found in sperm, such as Hsp70, PrxIII, Cox7c, and ATP1B1. The docking results showed a level of non-covalent interactions (NCIs) arising from the complexation between the receptors and the compound under study. Carvacrol showed the most favorable interaction with Cox7c (Fig. 4c), exhibiting the lowest binding energy of − 6.22 kcal/mol and the smallest inhibition constant Ki of 27.68 µM. Additionally, the binding energies of Hsp70 (Fig. 4a), PrxIII (Fig. 4b), and ATP1B1 (Fig. 4d) were − 4.93, − 4.44, and − 5.36, with inhibition constants Ki of 243.99, 554.1, and 117.51, respectively, when interacting with carvacrol molecules.

**Fig. 5:**
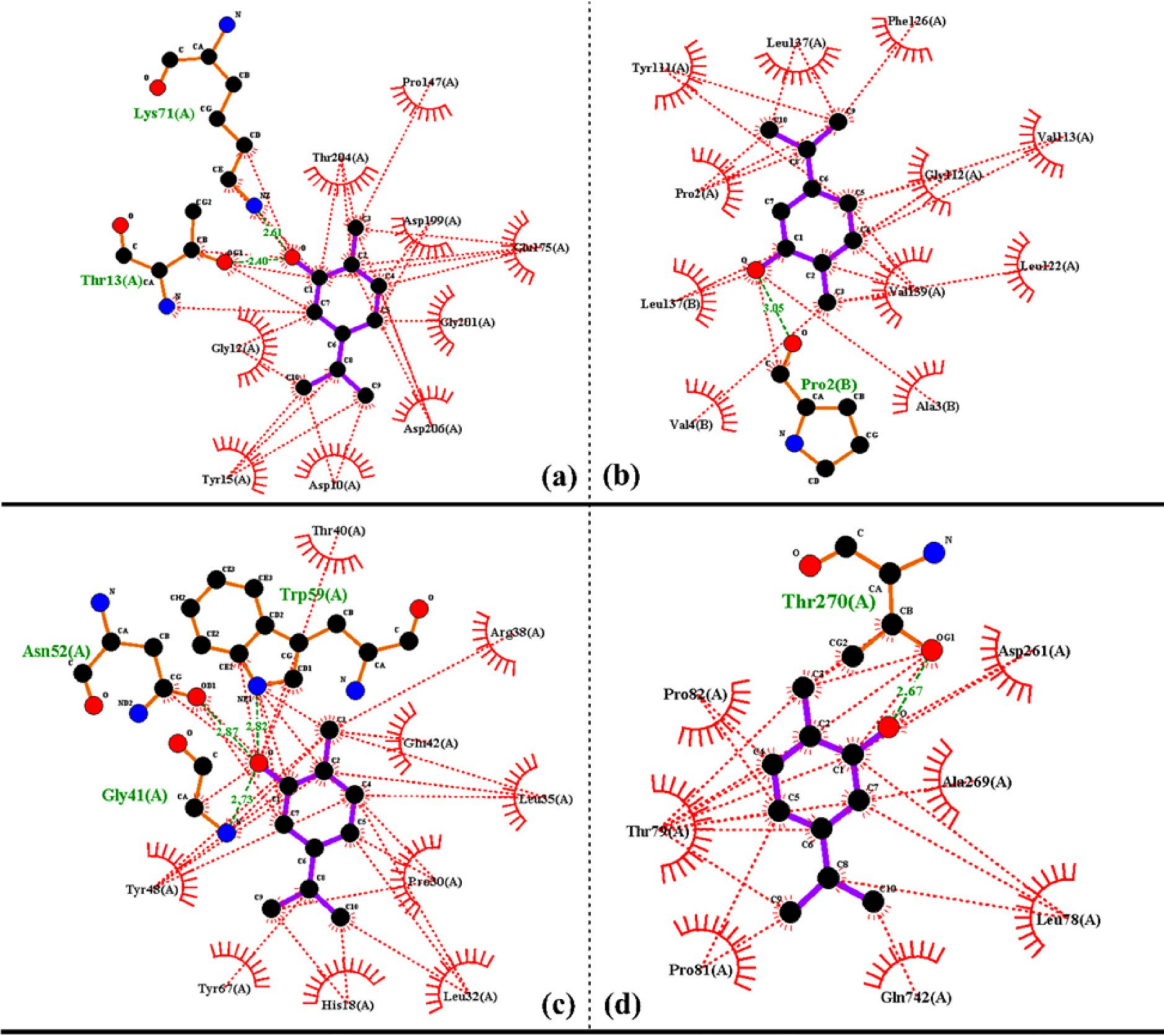
The hydrophobic interactions between carvacrol and different amino acids of protein receptors Hsp70 (**a**), PrxIII (**b**), Cox7c (**c**) and ATP1B1 (**d**) in buffalo sperm.

The hydrophobic interactions between carvacrol and various amino acids of protein receptors are illustrated in Fig. 5. The figure displays the optimal poses of the best conformers, along with 3D and 2D images depicting interactions between different amino acids of protein receptors Hsp70 (Fig. 5a), PrxIII (Fig. 5b), Cox7c (Fig. 5c), and ATP1B1 (Fig. 5d) with carvacrol molecules. It is worth noting that unfavorable repulsion was observed in the interaction between carvacrol and Hsp70 via the THR13 amino acid (Fig. 5a). Non-covalent interactions, specifically hydrogen bonding, van der Waals forces, and electrostatic interactions, were observed between carvacrol and the receptors. These interactions were influenced by the specific molecular groups involved, including alkyl, π-alkyl, π-anion, and amide π-stacking interactions. The presence of the hydroxyl group in carvacrol facilitated hydrogen bonding in all interactions with the receptors, as detailed in Table 9.

**Table 9:** The characterization of molecular docking study using carvacrol binding with some proteins (Hsp70, PrxIII, Cox7c and 4XE5) in sperm of buffalo bull.

Protein receptor	ID code	Binding energy (Kcal/mol)	Inhibition constant Ki (µM)	Reference RMSD	H-bonding
Hsp70	7O6R	− 4.93	243.99	23.42	Conventional H-bonding LIG H:THR13(A) O
PrxIII	4MH3	− 4.44	554.1	85.0	Conventional H-bonding LIG H:PRO2(B) O LIG O:VAL4(B) H
Cox7c	6FF5	− 6.22	27.68	20.73	Conventional H-bonding LIG H: ASN52(A) O LIG O:GLY41(A) H LIG O:TRP59(A) H Pi donor H-bonding LIG benzene ring:TYR48(A) H
ATP1B1	4XE5	− 5.36	117.51	60.87	Conventional H-bonding LIG H:ASP261(A) O LIG H:THR79(A) O

Heat shock protein family A (Hsp70), Mitochondrial 2-cys peroxiredoxin III (PrxIII), Cytochrome c oxidase subunit 7C (Cox7c), and ATPase Subunit Beta-1 (ATP1B1).

## Discussion

This study successfully synthesized carvacrol-loaded phytosomes (CLNPs) and demonstrated their efficacy in optimizing buffalo semen cryopreservation. Incorporating CLNPs into the freezing extender notably enhanced sperm quality, kinematic parameters, and antioxidant status, while concurrently reducing oxidative stress, microbiota load, and apoptosis. These in vitro benefits were supported by molecular docking analysis (In silico) and translated to improved conception rates (86% vs 72%, ex vivo). The inherent antioxidant activity of carvacrol, often limited by its poor water solubility and bioavailability, was effectively addressed through phytosome encapsulation. This lipid-based vesicular delivery system offers a food-grade approach to enhance the solubility and bioavailability of lipophilic compounds like CAR, highlighting its potential for developing novel drug and food formulations.

Optimizing sperm quality following cryopreservation is crucial for successful assisted reproductive technologies in animals. While antioxidants such as CAR have been investigated to mitigate OS and enhance sperm function in human^23^, the impact of CAR delivered via CLNPs on cryopreserved semen in animal studies remain largely unexplored.

This study demonstrated that a novel CAR formulation significantly improved sperm motility, viability, and membrane integrity in cryopreserved buffalo semen. Consistent with our findings, Cheraghi et al.^23^ showed that a 100 µM CAR supplement significantly increased sperm motility and viability in human semen freezing media. Additionally, carvacrol (200 µM) treatment significantly increased the progressive motility and viability of Beni Arouss buck semen compared to both control and thymol groups after 48 h of storage at 4 °C^24^. In agreement with our current research, serval studies have demonstrated that adding carvacrol did not affect sperm abnormalities^23^. The observed improvement in sperm motility could be attributed to CAR’s capacity to open ATP-dependent potassium channels and inhibit apoptosis^41^. However, the precise machinery through which CAR enhances sperm motility requires further investigation.

CASA is a versatile tool for assessing sperm motion characteristics. Cryopreservation induces significant changes in sperm motility, which consequently diminishes sperm function. In this study, we found that all CLNPs supplemented groups had higher PM, STR, DAP, DCL, VCL, DSL, and LIN. These parameters are critical indicators for assessing sperm motion. Consistent with our findings, Kchikich et al.^24^ reported that carvacrol significantly improved sperm motility parameters (VCL, VSL, and VAP) in buck semen stored at 4 °C for 48 h. Previous studies have indicated that nanoparticles can enhance sperm motility, as assessed by CASA^6^.

The addition of 100 µM carvacrol to human semen freezing media significantly increased total antioxidant capacity (TAC) and the levels of antioxidant enzymes, including catalase, glutathione, and superoxide dismutase^23^, underscoring the potent antioxidant properties of carvacrol. Additionally, that study found that carvacrol significantly reduced levels of MDA and DNA fragmentation compared to the control group.

Similarly, Gur et al.^42^ demonstrated that carvacrol can mitigate arsenic-induced oxidative stress by modulating autophagy and inflammatory pathways. This is supported by the upregulation of biomarkers such as LC3A, LC3B, MAPK-14, NF-κB, TNF-α, IL-1β, iNOS, and COX-2 in rat models. The findings regarding antioxidant parameters align with those of Shoorei et al.^43^, who reported that carvacrol enhanced antioxidant capacity and diminished the detrimental effects of diabetes-induced oxidative stress in a rat model. Consistent with these observations, supplementing cooled buck semen with carvacrol significantly reduced MDA levels after 48 h of storage at 4 °C (*P* < 0.05) compared to the control^24^.

Cryopreservation can induce various detrimental changes in sperm cells, such as membrane damage and cell death^44^, largely due to the overproduction of ROS. Elevated ROS levels have been linked to increased apoptosis and impaired sperm quality, which can ultimately contribute to male infertility^45^. Therefore, the inclusion of anti-apoptotic compounds may improve sperm function and quality during cryopreservation in animals.

Our findings demonstrated that treatment with CLNPs led to an increase in the number of viable sperm and a significant reduction in apoptosis, particularly within the concentration range of 5–20 µg/mL of extender. Consistent with this sense, previous research has shown that CAR treatment inhibited arsenic-induced apoptosis in the testes by downregulating the expression of Bax and Caspase-3, while upregulating Bcl-2 expression^42^. Similarly, carvacrol has been shown to significantly downregulate Bax and upregulate Bcl-2 at both the mRNA and protein levels, resulting in a significant decrease in germ cell apoptosis in diabetic rats^43^. These results align with our previous findings, which demonstrated that various nanoparticles enhanced sperm viability and reduced apoptotic and necrotic sperm populations following cryo-storage in buffalo, camel, rabbit, and ram semen^5,6,13,14^. While the anti-apoptotic properties of carvacrol have been corroborated by several studies^24,43^, further research is warranted to explore its potential advantages when incorporated into novel delivery systems such as CLNPs. Healthy mitochondria are essential for ATP production, which is critical for sperm motility and fertilization. Infertility can arise from mitochondrial dysfunction, often caused by cryopreservation procedures that may decrease mitochondrial DNA and elevate ROS levels^46^. A promising strategy to counteract the detrimental effects of cryopreservation and maintain mitochondrial structure is to supplement freezing media with mitochondrial enhancers. Our study indicates that the inclusion of CLNPs in buffalo bull semen freezing media can enhance MMP, potentially leading to improved fertilization capacity. This is supported by the increased conception rates observed in our study. Carvacrol (100 µM) has been shown to significantly enhance MMP in human semen freezing media^23^. Likewise, carvacrol significantly reduced ROS production and modulated mitochondrial activity in porcine semen cooled at 4 °C^47^.

Elevated semen bacterial populations can negatively impact sperm function. In this study, we observed that CLNPs significantly reduced the presence of pathogenic bacteria in cryopreserved semen. These findings align with those of a previous study^24^, which reported that CAR significantly decreased the bacterial load in buck semen stored at 4 °C for 4 h. This evidence supports the established antimicrobial efficacy of carvacrol. The presence of a phenol ring and a free hydroxyl group contributes to both the antioxidant and antimicrobial properties of CAR^10^.

The antibacterial activity of CAR molecules has been attributed to their significant effects on the structural and functional properties of the cytoplasmic membrane of bacteria. Mechanistically, their action is associated with the disruption of the bacterial cell membrane^48^. Other studies have indicated that carvacrol can reduce apoptosis via both intrinsic and extrinsic pathways or by scavenging oxidative markers due to the hydroxyl groups in its structure. CLNPs have also been used to improve intestinal health in broilers by modulating the gut microbiota, specifically by reducing pathogenic bacteria and increasing beneficial lactic acid bacteria^22^. These diverse actions highlight the multi-target potential of CAR when incorporated into nanotechnology-based approaches, yielding promising outcomes.

To validate our in vitro findings, we conducted ex-vivo experiments by inseminating buffalo cows with semen supplemented with 20 µg of CLNPs. The significantly higher pregnancy rates achieved with the CLNPs-treated semen compared to the control group confirmed the capacity of CLNPs to enhance sperm cryotolerance and improve sperm function. These in vivo results corroborate previous findings by Khalil et al.^6^ who reported a superior conception rate using thymoquinone nanoparticles compared to control semen in buffalo.

Ultrastructural analysis of sperm offers crucial insights into the damage caused by cryopreservation and the potential protective roles of CLNPs. Our study revealed that cryopreservation led to damage in the plasma membrane (complete), mitochondria, and acrosome in the control group and at lower CLNPs concentrations (2.5 and 5 µg/mL). However, higher CLNPs concentrations (10 and 20 µg/mL) seemed to provide a protective effect, as sperm cells at these concentrations displayed a normal nucleus, plasma membrane, mitochondria, and acrosome. These findings contrast with previous reports on buffalo sperm cryopreservation^5,6^. Several phytochemical-loaded nano-phytosomes, including alpha-lipoic acid-loaded nanoliposomes^49^, propolis-loaded nanoliposomes^5^, and curcumin-loaded niosomal nanocarriers^50^ have been utilized to reduce sperm damage induced by cryopreservation.

Given resource limitations that restricted molecular validation techniques (such as gene expression, transcriptomics, and proteomics) and western blotting analysis, we performed an in-silico study to explore the potential interactions of CLNPs with specific protein receptors on buffalo sperm, which could potentially stimulate molecular signaling pathways. This in silico study investigates the association between carvacrol and certain protein receptors present in sperm, including Hsp70, PrxIII, Cox7c, and ATP1B1. HSP70 is a key factor influencing sperm’s fertilization capacity, with high gene or protein expression levels of this molecule being linked to good semen quality.

Reddy et al.^51^ observed that the addition of sericin to the freezing extender of goat semen improved HSP70 levels in sperm cells. Our analysis revealed a significant binding affinity between CAR and HSP70, approximately − 4.93 kcal/mol. The strongest binding affinity was observed between CAR and Cox7c (− 6.22 kcal/mol), followed by ATP1B1 (− 5.36 kcal/mol). Cox7c levels were higher in bulls with high fertility, suggesting significant roles in sperm function^52^ and overall sperm fertility^53^. It has also been indicated as a potential predictor of fertility and the quality of post-thawed sperm function in bulls^54^. Cox7c is a protein involved in the final phase of the electron transport chain, which is responsible for ATP production in the inner mitochondrial membrane^55^. ATP1B1 mRNA expression in fresh sperm is considered an indicator of cryotolerance^56^. This protein, encoded by the ATP1B1 gene, belongs to the Na + /K + -ATPase pump family, which is crucial for maintaining the electrochemical gradient across cellular membranes^57^. This gradient can be disrupted by osmotic stress.

The ATP1B1 protein has been observed in the epididymal sperm of swamp buffaloes and bovine bulls, and its notably high presence in bull epididymal sperm has been correlated with high sperm freezability^58^. Mitochondrial 2-cys peroxiredoxin III (PrxIII) is a vital antioxidant enzyme that protects mitochondria by neutralizing harmful H_2_O_2_^59^.

This study demonstrated a strong binding affinity between CAR and PrxIII. This finding was further supported by the significant reduction in H_2_O_2_ levels observed when CLNPs were added to buffalo bull semen freezing extenders. Furthermore, Cao et al.^60^, suggested that this novel active-site organization could provide insights into the dynamic conformational adjustments between the oxidized and reduced states of the enzyme.

The pregnancy rate, also known as the non-return rate, is a reliable tool for indicating reproductive efficiency in livestock farms. Buffalo cows inseminated with thymoquinone nanoparticle-treated spermatozoa had a non-return rate of 90.9%^6^, while those inseminated with moringa leaves extract-treated spermatozoa had a rate of 88%^61^. These rates were higher than the respective control groups, which showed non-return rates of 54.55% and 72%. Consistent with our findings, study^13^ indicated that the addition of nano-emulsion essential oils from olive (76%), flaxseed (88%), and grape seed (92%) notably improved the non-return rate of buffalo semen compared to the control semen (68%). The observed improvement could be a consequence of CLNPs enhancing sperm quality and subsequently the fertility rate through their antioxidant and anti-apoptotic properties^9,32,43^.

## Conclusion

This study elucidated, for the first time, the application of phytosomes conjugated with carvacrol in enhancing cryopreserved buffalo semen. Our findings demonstrated that freezing medium supplemented with 10–20 µg/mL of CLNPs significantly improved sperm quality and kinematics, maintained sperm plasma membrane and acrosome integrity, and reduced oxidative stress biomarkers and apoptotic sperm. These in vitro results were supported by an increased pregnancy rate in buffalo cows inseminated with 20 µg/mL of CLNPs. Molecular docking simulations indicated that CAR exhibits a strong binding affinity for various sperm protein receptors, including Cox7c (− 6.22 kcal/mol), ATP1B1 (− 5.36 kcal/mol), HSP70 (− 4.93 kcal/mol), and PrxIII (− 4.44 kcal/mol). This novel phytomedicine delivery system presents new opportunities for assisted reproductive technologies to utilize innovative natural products for optimizing semen cryopreservation protocols. Future research should focus on further elucidating the precise molecular mechanisms underlying the observed interactions, as well as evaluating the applications of CLNPs in other relevant farm animals. This could broaden the potential of this promising natural remedy.

## Data Availability

The data supporting the findings of this study will be made available upon reasonable request to the corresponding authors.

## Abbreviations

CLNPs: Carvacrol-loaded phytosomes
MMP: Mitochondrial membrane potential
H_2_O_2_: Hydrogen peroxide
MDA: Malondialdehyde
TAC: Total antioxidant capacity
COX7C: Cytochrome C Oxidase Subunit 7C
Hsp70: Heat shock protein 70
PrxIII: Mitochondrial 2-cys peroxiredoxin III
ATP1B1: ATPase Na + /K + Transporting Subunit Beta 1
AI: Artificial insemination
OS: Oxidative stress
CAR: Carvacrol
PS: Particle size
PDI: Polydispersity index
ZP: Zeta potential
ROS: Reactive oxygen species
DSL: Distance straight line
DAP: Distance average path
DCL: Distance curved line
VAP: Velocity average path
VCL: Velocity curved line
STR: Straightness
VSL: Velocity straight line
LIN: Linearity
WOB: Wobble
BCF: Beat cross frequency (Hz)
ALH: Amplitude of lateral head displacement (µm)
